# Updates on the role of epigenetics in familial mediterranean fever (FMF)

**DOI:** 10.1186/s13023-024-03098-w

**Published:** 2024-02-26

**Authors:** Ahlam Chaaban, Zeina Salman, Louna Karam, Philippe Hussein Kobeissy, José-Noel Ibrahim

**Affiliations:** Department of Natural Sciences, School of Arts and Sciences, Lebanese American University (LAU), Beirut, Lebanon

**Keywords:** Familial Mediterranean Fever (FMF), Epigenetics, DNA methylation, Histone modification, miRNA

## Abstract

Familial Mediterranean Fever (FMF) is an autosomal recessive autoinflammatory disease caused by mutations in the *MEFV* (*ME*diterranean *F*e*V*er) gene that affects people originating from the Mediterranean Sea. The high variability in severity and clinical manifestations observed not only between ethnic groups but also between and within families is mainly related to *MEFV* allelic heterogeneity and to some modifying genes. In addition to the genetic factors underlying FMF, the environment plays a significant role in the development and manifestation of this disease through various epigenetic mechanisms, including DNA methylation, histone modification, and noncoding RNAs. Indeed, epigenetic events have been identified as an important pathophysiological determinant of FMF and co-factors shaping the clinical picture and outcome of the disease. Therefore, it is essential to better understand the contribution of epigenetic factors to autoinflammatory diseases, namely, FMF, to improve disease prognosis and potentially develop effective targeted therapies. In this review, we highlight the latest updates on the role of epigenetics in FMF.

## Introduction

The first proper description of Familial Mediterranean Fever (FMF) was coined in 1945 by Siegel as ‘benign paroxysmal peritonitis’. However, it was not until a decade later that Heller provided the universal name ‘Familial Mediterranean Fever’, which has since been in use [1]. FMF is a disease that mainly affects certain populations originating from the Eastern Mediterranean, such as Arabs, Armenians, and Turks [2]; however, due to increased immigration, FMF has become prevalent worldwide [3], with hundreds of Japanese, North American, and European people reporting cases of FMF [2]. Among the populations affected by FMF, Turks rank first, with a prevalence ranging between 1:150 and 1:1,000, followed by Armenians, with a prevalence reported to be 1:500 [4]. In addition, there are a significant number of FMF cases in Arabic countries such as Lebanon and Jordan; however, exact statistics have not yet been reported [5].

FMF is a genetic disease caused by mutations in the *MEFV* (*ME*diterranean *F*e*V*er) gene, which is present on the short arm of chromosome 16 (16p13.3) and is made of 10 exons [6, 7]. According to the "Infevers" database, there are currently 396 variants of the *MEFV* gene. Most of these variants are located in exons 2 and 10, and approximately 96% of them are substitutions. These variants are classified as pathogenic, likely pathogenic, benign, likely benign, or variants of uncertain significance (VUS) [8]. The most common mutations in the *MEFV* gene are M680I, M694V, M694I, and V726A, which are located in exon 10, along with the E148Q variant located in exon 2 [6, 7]. These variants are responsible for 85% of FMF patients worldwide [9]. Although M694V is considered to be the most common mutation worldwide [10, 11], the mutation frequency varies among countries, regions, and ethnic groups.

FMF is commonly inherited in an autosomal recessive manner [12]. Therefore, to be genetically diagnosed with FMF, a patient should present two mutations in the *MEFV* gene. However, another pattern of FMF inheritance, pseudodominance, was reported, particularly in countries where consanguineous marriages are common [13]. Furthermore, true autosomal dominant inheritance also occurs in some rare cases. In the study conducted by Booth et al. (2000), the *MEFV* gene was screened in five families whose inheritance seemed to be autosomal dominant [14]. Interestingly, three of these mutations were proven to be true dominant, and the patients were heterozygous for the delta M694 variant or compound heterozygous E148Q/M694I [14]. Similarly, Rowczenio et al. (2020) reported a novel *MEFV* variant, p.P373L, which was identified in four generations of a British family and showed a dominant transmission pattern with amyloidosis complications [15].

The clinical manifestations of FMF are broad and range in severity; the most common manifestations include fever, abdominal pain, arthritis, and thoracic pain. Some patients may develop amyloidosis at later stages, which is the most severe complication of FMF [16]. The clinical variability of the disease is mainly related to the allelic heterogeneity of the *MEFV* gene, with M694V exhibiting the highest penetrance and being associated with the most severe phenotype of the disease. In contrast, the clinical significance of E148Q has still not been determined. While some researchers support the idea that E148Q is a pathogenic variant with low penetrance, others tend to consider it a benign polymorphism [13]. Other factors contributing to the variable expressivity of the disease include modifying genes such as *IL-1β* [17, 18] and serum amyloid A1 (*SAA1*) [19]. Interestingly, numerous studies have recently shown that along with genetic susceptibility, epigenetic events are important pathophysiological factors contributing to the clinical manifestations of this disease in patients [20]. These epigenetic mechanisms can be affected by various environmental factors, such as stress, diet, and physical activity [21].

**Fig. 1:**
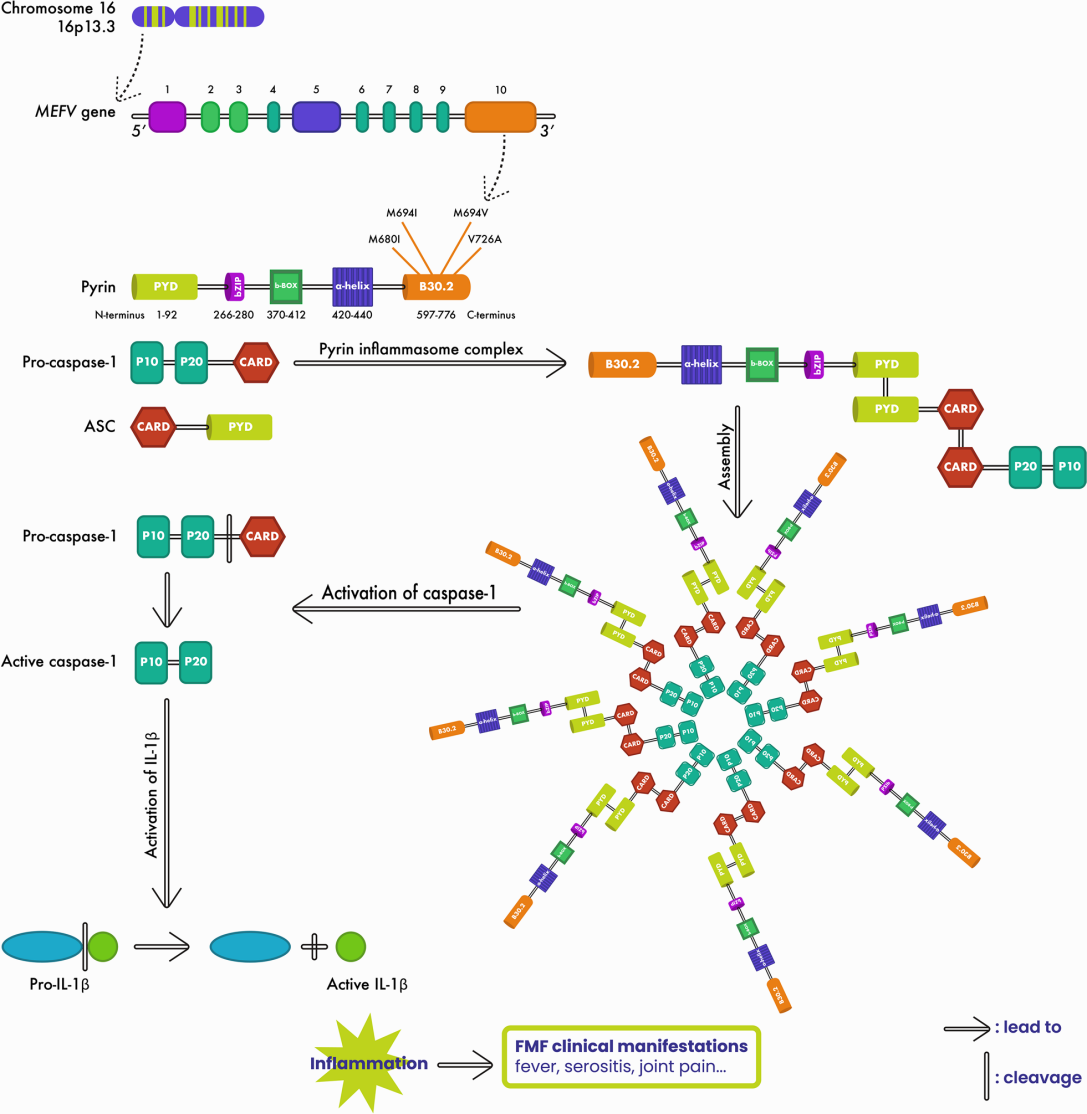
Pyrin inflammasome activation in FMF. *MEFV* gene, located on the short arm of chromosome 16 (16p 13.3), is made of 10 exons and encodes the pyrin protein. Pyrin is composed of the five domains PYD, bZIP transcription factor, B-box, α-helical coiled-coil, and B30.2 domains. The C-terminal domain, B30.2, is the most important domain where the most common FMF mutations (M680I, M694I, M694V, V726A) are clustered. *MEFV* mutations activate pyrin which form with pro-caspase-1 and ASC a multiprotein complex called inflammasome. The assembly of the pyrin inflammasome initiates autocatalytic activation of caspase-1 which in turn cleaves and converts pro-IL-1β to active IL-1β leading to inflammation and contributing to FMF clinical manifestations (fever, serositis, joint pain …). *MEFV*: MEditerranean FeVer; ASC: Apoptosis-associated speck-like protein, bZIP: Basic leucine zipper; CARD: Caspase recruitment domain; IL-1β: Interleukin-1 beta; PYD: Pyrin domain

The *MEFV* gene encodes a 95 kDa protein composed of 781 amino acids and is named pyrin or marenostrin [22]. This protein is expressed mainly in immune cells, such as granulocytes, eosinophils, monocytes, and dendritic cells [22]. It is composed of five distinct domains: the pyrin domain (PYD), bZIP transcription factor domain, B-box zinc finger domain, α-helical coiled-coil domain, and B30.2 domain [22]. The B30.2 domain, located at the C-terminus, is the most important domain of the pyrin protein because it harbors the most frequent and pathogenic FMF-associated mutations [7]. Pyrin, along with pro-caspase-1 and apoptosis-associated speck-like protein (ASC), is part of a multiprotein complex called inflammasome. Upon *MEFV* mutations, the pyrin inflammasome is assembled, leading to the activation of caspase-1 which in turn activates the cleavage of pro-IL-1β and secretion of mature IL-1β [23, 24]. Increased IL-1β production results in a subclinical inflammation in FMF patients even during asymptomatic periods [24, 25] (Fig. 1). Therefore, although colchicine has been used as the gold standard treatment for FMF since the 1970s [26], colchicine-resistant patients are administered IL-1β inhibitors such as anakinra (an IL-1 receptor antagonist) and canakinumab (a human monoclonal anti-IL-1β antibody) [27, 28].

With so many factors contributing to the pathogenesis of the disease, we can conclude that FMF is not a simple monogenic disease with a straightforward genotype‒phenotype correlation. In addition, pyrin activity is affected not only by *MEFV* mutations but also by epigenetic factors.

## Epigenetics

For a long time, it was thought that the only way to possibly achieve phenotypic variation was by modifying the DNA sequence. However, in 1942, Waddington introduced the term “epigenetics” [29], which currently refers to the study of changes that occur in the functions of genes that can be passed on to future generations without any direct changes in the DNA sequence itself [30].

Studying epigenetics aids in identifying different disease biomarkers, allowing researchers and health professionals to detect a certain disease, depending on the epigenetic mechanism linked to it, at an early stage before it has progressed or manifested. This approach helps in developing specific treatment or prevention plans for possible patients [31].

Multiple epigenetic mechanisms have been studied, with DNA methylation, histone modification, and noncoding RNAs, mainly microRNAs (miRNAs), being the most intensively investigated in the context of inflammatory diseases [32]. For this purpose, this review will focus on the role of these three mechanisms in FMF pathogenesis.

## DNA methylation

DNA methylation is the most common epigenetic mechanism. This process involves the addition of a methyl group from S-adenosyl-L-methionine (SAM) by DNA methyltransferases (DNMTs) to the fifth position of a cytosine to form 5-methylcytosine (5mC) [33], thus leading to changes in function without directly manipulating the DNA sequence itself. Most DNA methylation occurs in regions known as CpG islands, which are located mainly within promoters of different genes, hence leading to a decrease in the expression levels of the respective genes [34]. Upon the methylation of CG regions, the DNA adopts a packed conformation, which typically manifests as chromatin remodeling [35]. This prevents the transcriptional machinery from binding to promoters, thus preventing the expression of genes and decreasing their respective protein levels.

The *MEFV* gene has a 998-base pair (bp) CpG island that covers a part of the first intron and the entire exon 2 of the gene [36]. In this respect, it is expected that methylation of the *MEFV* gene might lead to phenotypic variability, and patients with a methylated variant of *MEFV* exon 2 may exhibit a more severe form of FMF [33]. However, this hypothesis was not confirmed due to inconsistencies in findings linking *MEFV* methylation and disease severity. For instance, a study conducted by Ulum et al. (2015) on Turkish children diagnosed with FMF revealed that there is no correlation between the methylation of the *MEFV* gene and the clinical symptoms presented by the patients [37]. The same research showed that patients homozygous for the M694V mutation had lower *MEFV* gene expression than patients heterozygous for the same mutation, thus suggesting the involvement of other epigenetic factors in the clinical picture of FMF. In another study performed in Turkey, pediatric FMF patients exhibited significantly lower levels of *MEFV* expression as compared to controls, but no correlation was found between *MEFV* methylation and *MEFV* expression [38]. These findings contrast with those of Kirectepe and colleagues (2011), who showed that methylation of the *MEFV* gene decreases its expression [36]. These differences in results might be attributed to the study design, sample size, and patient characteristics. Moreover, it is important to note that the mechanism of alternative splicing was investigated in the study conducted by Kirectepe et al. (2011) but not by Doğan et al. (2019). Indeed, Kirectepe et al. (2011) observed higher levels of splicing and methylation of exon 2 in FMF patients than in healthy controls, suggesting a link between these events and a decrease in gene expression. The methylation of exon 2 was associated with increased spliced transcripts of exon 2 (*MEFV-d2*) and abnormal localization of the d-2 pyrin protein in the nucleus instead of the cytoplasm, where the full-length pyrin protein is normally found. This disrupted function may contribute to the pathogenesis of FMF [39]. Additionally, FMF patients exhibited higher levels of d-2 pyrin compared to healthy individuals, providing further evidence of its potential role in FMF pathogenesis [36, 39].

Given the involvement of pyrin in the inflammasome complex and the established connection between the NLRP13 inflammasome and the *MEFV* gene, researchers were intrigued to compare *NLRP13* gene expression and methylation between FMF patients in crisis and healthy controls. Interestingly, the results revealed that the NRLP13 inflammasome is epigenetically regulated in FMF patients. In fact, the presence of amyloidosis was associated with hypermethylation of the *NRLP13* gene, hence resulting in its silencing and subsequently in the inhibition of inflammasome assembly. This discovery unveiled a new epigenetic mechanism contributing to the development of FMF [40]. The NLRP3 inflammasome was also found to be significantly demethylated in monocytes derived from FMF patients and associated with increased expression of IL-1β [41].

DNA methylation might also be involved in patients’ response to colchicine treatment. In a study conducted in 2023, the role of epigenetic modifications was investigated in a cohort of 63 FMF patients divided according to their responsiveness to colchicine and 19 healthy individuals. While the results showed that the colchicine nonresponders had a greater methylation level of exon 2 of the *MEFV* gene than did the colchicine responders, these results were deemed to be nonsignificant. As such, it was difficult to reach a conclusion regarding the effectiveness of colchicine in patients suffering from methylation, and additional studies need to be conducted to confirm this observation [33].

## Histone modification

Histone modification is another key epigenetic mechanism. Interestingly, DNA methylation and histone modification are highly interrelated within the context of chromatin modulation, and they affect each other throughout growth. While histone methylation mediates proper DNA methylation patterns, histone modification patterns are recreated using DNA methylation as a template after DNA replication [42].

Histone modifications include methylation, acetylation, phosphorylation, ubiquitination, and SUMOylation. They regulate gene expression either directly through altering chromatin structure or via protein adaptors termed effectors [43].

Like DNA methylation, histone methylation requires histone methyltransferases (HMTs) to transfer a methyl group from the SAM to the lysine and arginine groups of the histone [43]. Histone methylation is commonly linked to transcriptional repression; however, the outcome of gene expression can vary based on the specific methylation of lysine or arginine residues, leading to either gene activation or repression. For example, the methylation of H3K9, H3K27, and H4K20 leads to the repression of gene expression, while the methylation of H3K4, H3K36, and H3K79 is associated with the activation of gene transcription [44].

Histone acetylation has an opposite effect to histone methylation. It activates gene expression by neutralizing the positive charge of lysine residues, thus decreasing the affinity of histones for binding to DNA and allowing the DNA to remain accessible to the transcriptional machinery [45]. On the other hand, histone phosphorylation is mediated by protein kinases and occurs on serine, threonine, and tyrosine residues. It is most commonly associated with DNA repair and generally results in the activation of gene expression, similarly to acetylation [45].

Histone ubiquitination is performed via histone ubiquitination ligases, while histone deubiquitinating enzymes (DUBs) are responsible for reversing ubiquitination. Histone ubiquitination can lead to either the activation or repression of gene expression. The monoubiquitylation of H2A and H2B yielded the clearest results regarding the transcriptional outcome of genes. For example, the monoubiquitylation of H2A leads mainly to the repression of gene expression, while that of H2B results in the activation of gene expression (45). Finally, SUMOylation, which involves the covalent addition of a small ubiquitin-like modifier (SUMO) to a specific lysine, is generally associated with transcriptional repression [46].

Histone modifications have been found to play a role in the activation of the NLRP3 inflammasome and subsequently in the manifestation of multiple autoimmune and autoinflammatory disorders, such as systemic lupus erythematosus [47], rheumatoid arthritis [48], Behçet disease (BD) [49], atherosclerosis, and Alzheimer’s disease [50]. Nevertheless, to date, no study has investigated the role of histone modifications in FMF, despite the relevant implication of the NLRP3 inflammasome in the pathogenesis of this disease.

Histone acetylation dynamics play a major role in the regulation of the NLRP3 inflammasome. For instance, in a murine model of painful neuropathy, the acetylation of histones H3K9 and H4 in the promoter region of *NLRP3* increased its expression levels and activated the inflammasome [51]. Similarly, histone demethylation was shown to regulate the NLRP3 inflammasome. Specifically, KDM4B, a histone demethylase lysine-specific demethylase, was found to demethylate H3K9me3 in the *NLRP3* promoter region, upregulating *NLRP3* gene expression and promoting the inflammatory response [52].

BD shares many genetic and inflammatory features with FMF. In fact, BD and FMF might occur in the same individual more commonly than expected [53]. Additionally, *MEFV* mutations, particularly M694V, which activate the inflammasome complex, have been shown to increase the risk of BD in regions where both FMF and BD are prevalent [54, 55]. Therefore, it will be of particular interest to review and understand how histone modifications might affect BD manifestation. In vitro activation of Sirtuin 1 (SIRT1), which is a histone deacetylase, in normal human and ocular BD donor PBMCs suppressed T-cell proliferation and proinflammatory cytokine production [56]. Similarly, Xie and Yang (2021) observed a decrease in *SIRT1* expression in CD4 + T cells obtained from patients diagnosed with active ocular BD. Interestingly, the activation of SIRT1 inhibited Th17 and Th22 cell proliferation and significantly suppressed the secretion of IL-17 and IL-22 [57]. Accordingly, it was suggested that the regulation of histone acetylation, especially the activation of SIRT1, is a potential treatment for BD-related uveitis.

Ubiquitination and SUMOylation have also been studied in the context of BD. For instance, the ubiquitination-related genes *UBAC2* and *UBASH3B* were found to be associated with susceptibility to BD in many populations [58, 59]. On the other hand, the genetic association between *SUMO4* polymorphisms and BD was first reported among Chinese Han patients [60]. The results were replicated in Tunisian and Korean cohorts, in which specific polymorphisms were linked to BD severity and the development of skin lesions [61, 62]. Although the functions of the proteins encoded by *UBAC2* and *UBASH3B* are still unclear, SUMO4 is believed to participate in BD pathogenesis via the regulation of NF-ĸB signaling pathways and the expression of proinflammatory cytokines.

Given the shared pathophysiological features between FMF and BD, exploring similar mechanisms in different experimental models of FMF can help us gain more insights into the role of epigenetics in disease expression and manifestation [63].

## miRNAs

Another common process by which gene expression is modified without directly affecting sequence is through miRNAs. miRNAs are small noncoding RNA molecules with an average length of 18–25 nucleotides that regulate gene expression through RNA interference mechanisms such as mRNA chopping, mRNA deadenylation, and translation inhibition [64]. They can also promote gene expression by activating translation and regulating transcription. The affinity of miRNA‒mRNA interactions, the abundance of miRNAs and target mRNAs, and the subcellular localization of miRNAs are only few of the variables that affect how miRNAs interact with their target genes to regulate gene expression (64).

In addition to their intracellular role, miRNAs can be secreted into extracellular fluids and delivered to target cells by attaching to proteins or vesicles, such as exosomes, being transported without vesicles*,* or being carried by high-density lipoprotein in peripheral blood [65]. These extracellular miRNAs serve as chemical messengers that facilitate communication between cells [66]. In humans, approximately 2600 mature miRNAs have been reported in the MiRbase (release 22.1) [67, 68] to control the expression of approximately 60% of protein-coding genes [69]. It is important to highlight that hundreds of mRNAs can be the targets of one miRNA; hence, simultaneously, a single miRNA affects the expression of many genes that tend to be engaged in interacting pathways [70].

Gene regulation by miRNAs begins in the cytoplasm once they bind to mRNAs. Upon binding, miRNAs reduce gene expression by suppressing mRNA translation, after which mRNAs are either degraded or recycled or can be saved for later translation. In addition to translation repression, miRNA can reduce gene expression by mRNA deadenylation and decapping upon binding to a specific sequence in the 3' untranslated region (UTR) [71, 72]. However, the binding of a miRNA to its target mRNA is not restricted to this region. Several studies have recognized sequences in the 5' UTR and within the promoter as binding sites for miRNAs as well [73]. Like in the 3’UTR, miRNA binding to the 5’ UTR sequence causes gene silencing [74, 75], while binding to the promoter region seems to promote transcription and gene expression [76].

miRNAs are known to be important modulators of inflammation [77]. A number of immune response mechanisms, including the growth and differentiation of B and T cells, the expansion of monocytes and neutrophils, the activation of antibodies, and the release of inflammatory regulators, have been linked to miRNAs [78]. In fact, the involvement of miRNAs in inflammation is broad and complex. miRNAs regulate both proinflammatory and anti-inflammatory processes. For instance, hsa-let-7d-5p inhibits the proliferation of Th1 cells and interferon (IFN)-γ secretion [79]. In contrast, miR-23a promotes inflammation and proinflammatory cytokine synthesis by activating the NF-ĸB signaling pathway and inhibiting the anti-inflammatory JAK1/STAT6 pathway [80]. In turn, the expression of miRNAs may be upregulated by inflammatory cytokines such as tumor necrosis factor (TNF)-α and IFN-γ.

On the other hand, several studies have proposed a role for miRNAs in regulating inflammasome activation in two ways. The first is by directly targeting inflammasome components. For example, many studies have reported that miR-223 is considered to be the direct target of NLRP3 [81–83], while miR-142 is the target of ASC [84] and that miR-446 is the direct target of pro-IL-1β [85]. The other way may be through monitoring the function of inflammasome regulators. In fact, miR-146a [75] and miR-96 [86] are associated with NF-ĸB pathway regulation, miR-33 targets reactive oxygen species (ROS) [87], and miR-17 [88] and miR-20a [89] are involved in thioredoxin-interacting protein (TXNIP) production [90].

The role of miRNAs in FMF has received increasing attention, and there is a growing body of evidence on the role of miRNAs in different mechanisms involved in FMF pathogenesis, such as apoptosis, inflammation, and autophagy [91, 92]. In 2018, Amarilyo et al. evaluated the expression levels of miRNAs in the PBMCs of 10 FMF patients homozygous for the M694V mutation, in remission, and under colchicine treatment, and 10 healthy subjects [93]. The study revealed that 7 miRNAs were differentially expressed in FMF patients; miR-144-3p, miR-21-5p, miR-4454, and miR-451 had higher expression levels in patients than in controls; and miR-107, let-7d-5p, and miR-148b-3p were downregulated in patients compared to controls [93]. However, Wada et al. (2017) reported no significant difference in the expression of these miRNAs, with the exception of miR-451a, which was also found to be downregulated in the serum of FMF patients [94]. Such variations may be due to differences in sample size and characteristics, biological samples tested, and quantification methods.

Interestingly, all the miRNAs studied by Amarilyo et al. (2018) are involved in immune processes. In fact, miR-148-3p, which was shown to be downregulated in FMF patients [93], is known to have an anti-inflammatory effect by inhibiting the production of the cytokines IL-12, IL-6, and TNF-α [95]. On the other hand, miR-144-3p is upregulated in FMF patients in remission [93] and is known to have a proinflammatory effect, as it was found to be positively associated with IL-1β levels in lung cancer patients [96]. These results support the findings of Ibrahim et al. (2014), who highlighted the presence of ongoing subclinical inflammation even in asymptomatic periods, particularly among M694V homozygous patients [17].

Another study conducted by Kahraman et al. in 2021 aimed to evaluate the role of a subset of 13 miRNAs in relation to immune functions in 28 diagnosed FMF patients and 28 controls from Turkey [97]. The results revealed the overexpression of miR-34a-5p, miR-142-3p, miR-216a-5p, miR-340-5p, miR-429, and miR-582-5p and the downregulation of miR-107, miR-569, and miR-1304-5p in FMF patients compared to those in controls. Furthermore, significantly lower expression levels of miR-107 were detected in patients homozygous for the M694V mutation than in patients carrying other mutations. Accordingly, the authors suggested a possible association between miR-107 and the M694V genotype and the potential use of this particular miRNA as a therapeutic and diagnostic tool for FMF [97].

**Fig. 2:**
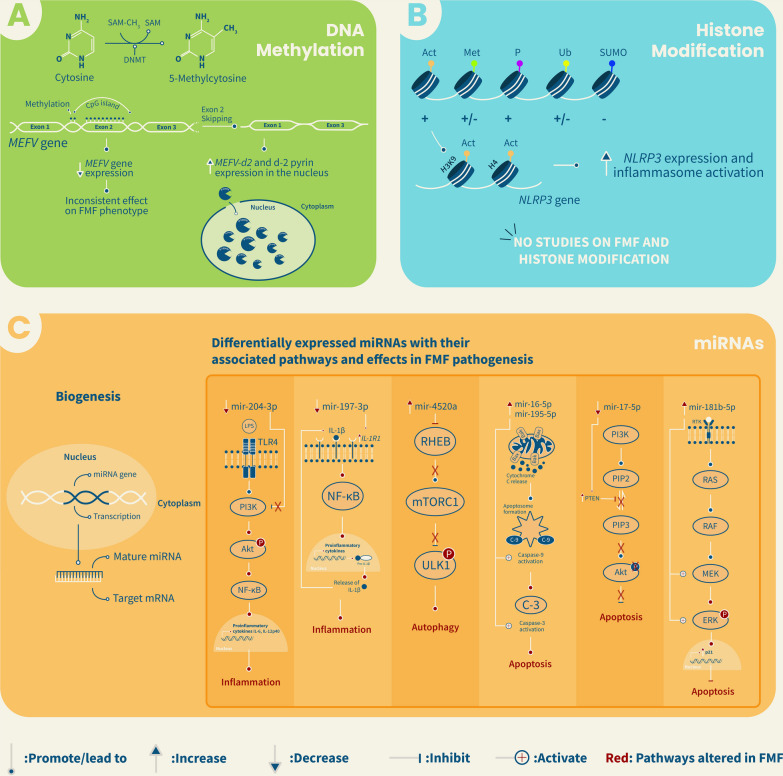
Schematic representation of key epigenetic mechanisms (DNA methylation, histone modification, and miRNA) involved in the pathogenesis of Familial Mediterranean Fever (FMF). (**A**) DNA methylation of the CpG island covering the entire exon 2 of *MEFV* gene leads to a decrease in the gene expression and to exon 2 skipping, hence resulting in an increase in spliced transcripts of exon 2 (*MEFV-d2*) and an abnormal localization of d-2 pyrin protein in the nucleus. Such changes may contribute to FMF pathogenesis and phenotypic variability among patients. (**B**) Acetylation of H3K9 and H4 histones of the *NLRP3* gene promoter results in an increase of *NLRP3* gene expression and inflammasome activation. The role of histone modifications in FMF pathogenesis has not yet been elucidated. (**C**) miRNAs, such as mir-204-3p, mir-197-3p, mir-4520a, mir-16-5p, mir-195-5p, mir-17-5p, mir-25, and mir-181b-5p, present variable expression in FMF patients. These miRNAs are involved through various signaling pathways in many processes such as inflammation, autophagy, and apoptosis. For example, the downregulation of mir-204-3p in FMF was found to activate PI3K leading to the phosphorylation of Akt which in turn activates NF-κB that promotes the expression of pro-inflammatory cytokines such as IL-6 and IL-12p40, triggering inflammation. Similarly, the downregulation of mir-197-3p in FMF leads to inflammation by increasing the expression of IL-1R1, activating downstream NF-κB which promotes the expression and release of IL-1β. Moreover, the upregulation of mir-4520a in FMF patients was found to further inhibit *RHEB*, inactivating the mTORC1 pathway and promoting the phosphorylation of ULK1 which induces autophagy. Overexpression of both mir-16-5p and mir-195-5p in FMF patients leads to the activation of caspase-9 and caspase-3, inducing apoptosis. As for mir-17-5p, its down-expression in FMF was found to increase the expression of PTEN which inhibits the conversion of PIP2 to PIP3, preventing the phosphorylation of Akt, and inducing apoptosis. Finally, the upregulation of mir-181b-5 activates MEK and ERK, hence increasing the expression of *p21* and repressing apoptosis. Act: Acetylation; Akt: Protein kinase B; DNMT: DNA methyltransferases; ERK: Extracellular signal-regulated kinase; IL: Interleukin; IL1-R1: Interleukin-1-receptor-1; LPS: Lipopolysaccharide; MEFV: MEditerranean FeVer; MEK: Mitogen-activated protein kinase; Met: Methylation; mTOR: Mammalian target of rapamycin; NF-κB: Nuclear factor kappa-light-chain-enhancer of activated B cells; P: Phosphorylation; PIP2: Phosphatidylinositol 4,5-bisphosphate; PIP3: Phosphatidylinositol 3,4,5-triphosphate; PI3K- γ: Phosphoinositide 3-kinases gamma; PTEN: Phosphatase and tensin homolog; RAF: Rapidly accelerated fibrosarcoma; RAS: Rat sarcoma; RHEB: Ras homolog enriched in brain; SAM: S-Adenosylmethionine; SUMO: SUMOylation; TLR4: Toll-like receptor 4; Ub: Ubiquitylation; ULK1: Unc-51 like autophagy activating kinase 1

Koga et al. (2017) analyzed the serum of Japanese FMF patients in the quiescent and attack phases and revealed that miR-204-3p was downregulated in patients during attacks compared to controls and patients in remission [98]. Similar findings were observed in the study of Demir et al. (2020) involving 30 FMF children from Turkey and 30 healthy children, in which a significant 6.5-fold reduction in miR-204 expression was detected in patients’ plasma during crises in comparison to controls and to children during remission periods [99]. miR-204-3p was shown to target the phosphoinositide 3-kinase gamma (PI3Kγ) pathway, which usually promotes the release of inflammatory cytokines, including IL-12p40 and IL-6 [98]. Hence, researchers proposed the use of miR-204-3p as a potential therapeutic tool to inhibit the PI3Kγ pathway in FMF patients and reduce inflammation [98] (Fig. 2).

Moreover, the microarray results of Latsoudis et al. (2017) revealed higher expression levels of miR-4520a in 9 FMF patients from Crete than in healthy individuals. In addition, they reported an increase in the expression of miR-4520a in a human premonocytic cell line (THP-1) lacking the *MEFV* gene and showed that miR-4520a targets RHEB, which activates the mTOR pathway involved in autophagy [100] (Fig. 2). Therefore, the authors suggested that autophagy is involved in FMF pathogenesis through the deregulation of miR-4520 expression dependent on the presence of *MEFV* mutations [100].

In 2017, Akkaya-Ulum et al. revealed that several miRNAs involved in inflammatory pathways are differentially expressed in FMF patients harboring one or two *MEFV* mutations compared to controls and healthy carriers; moreover, miR-20a-5p and miR-197-3p were overexpressed and downregulated, respectively, in homozygous patients, and let-7d-3p and miR-574-3p expression levels were increased in heterozygotes [91]. In silico analyses revealed that all four miRNAs interfered with the TGF-β (Transforming growth factor beta) signaling pathway, the TLR (Toll-like receptor) and NLR (NOD-like receptor) signaling pathways, apoptosis, and actin cytoskeleton regulation [91]. Interestingly, it was noted that miR-197-3p expression levels varied with regard to patient treatment; all homozygous patients were receiving biologic drugs such as anakinra and canakinumab; however, heterozygous patients were treated with colchicine [91]. This distinct miRNA profile may help to better understand the phenotypic variability observed among FMF patients.

In 2021, the same group of researchers thought to further detail the role of miR-197-3p in FMF [101]. By transfecting THP1 cells with pre-miR-197, miR-197-3p was proven to have an anti-inflammatory effect because the overexpression of miR-197-3p led to a decrease in the expression of the *IL-1β, TNF-α*, and *TGF-β* genes. Moreover, using a 3’UTR luciferase activity assay, it was demonstrated that miR-197-3p targets *IL-1R1*. miR-197-3p represses IL-1R1 expression on the membrane, which leads to a decrease in the activation of the NF-ĸB signal transduction pathway and a reduction in the expression of the IL-1β precursor (Fig. 2). In this respect, the authors suggested that increasing miR-197-3p expression in FMF patients, would be an interesting approach to treat FMF patients, especially those who are refractory to colchicine [101].

On the other hand, 15 miRNAs known to be involved in autoinflammatory diseases and immune responses were evaluated in FMF patients treated with colchicine [102]. After observing reduced expression levels of miR-125a, miR-15a, miR-132, miR-155, miR-146a, miR-26a, miR-16, miR-223, miR-181a, miR-21, and miR-34a in patients in comparison to controls, the authors compared the levels between treated and untreated patients. Interestingly, FMF patients treated with colchicine had significantly greater levels of miR-132, miR-15a, miR-181a, miR-23b, and miR-26a than did untreated patients, suggesting a potential correlation between colchicine and the expression of these miRNAs [102]. The exact mechanism of action of the majority of these miRNAs has not yet been elucidated; however, it has been reported that mir-155 plays an anti-inflammatory role by inhibiting molecules, including matrix metalloproteinases, protein phosphatase 2A, TLR ligands, and IL-2 [103]. Similarly, like NF-κB kinase inhibitors, mir-223, mir-16, and mir-15a have been shown to decrease inflammation by inhibiting the NF-κB pathway [104].

In addition to being involved in inflammation, miRNAs were suggested to play a role in FMF progression by affecting apoptotic pathways. In fact, a 2021 study conducted by Karpuzoglu and colleagues analyzed 33 miRNAs involved in apoptosis in 191 FMF children and 31 controls from Turkey. Researchers have shown that 26 miRNAs are differentially expressed in FMF children, with 19 miRNAs being upregulated and seven downregulated [105]. The correlation between apoptosis and FMF pathogenesis is not straightforward. For instance, miR-16-5p and miR-195-5p, which are upregulated in FMF patients, are suggested to activate caspases 3 and 9, which promote apoptosis [106, 107]. Moreover, miR-181b-5p, which was found to be overexpressed in FMF patients, represses apoptosis by affecting the MEK/ERK/p21 pathway [108] (Fig. 2).

Finally, the use of miRNAs as biomarkers was investigated recently by Abdelkawy et al. in 2021 [109]. This study was conducted on 50 FMF patients receiving colchicine treatment and 25 controls from Egypt to assess the plasma expression levels of miR-181a and miR-125a and the levels of the proinflammatory cytokines IFN-γ and IL-17. Compared with healthy controls, FMF patients expressed higher levels of IL-17 and IFN-γ and lower levels of miR-181a and miR-125a. However, there was no significant association between cytokine levels and the expression of the studied miRNAs. miR-181a is known to be involved in the differentiation and activation of T cells [110]. Indeed, Abdelkawy et al. (2021) [109], Li et al. (2007) [111], and Schaffert et al. (2015) [112] reported a positive correlation between miR-181a and lymphocyte percentage. Moreover, miR-125a has been shown to have an anti-inflammatory role in inflammatory diseases, mainly rheumatoid arthritis and Crohn’s disease [113, 114], by decreasing the levels of the IL-10 receptor α, the IL-2 receptor β, and IFN-γ [109]. The anti-inflammatory role of miR-125a was further supported by the findings of Abdelkawy and colleagues who showed a negative correlation between miR-125a and C-reactive protein in FMF patients [109]. Therefore, various miRNAs, particularly miR-181a and miR-125a, may be used as biomarkers of inflammation in FMF patients.

Table 1 summarizes the different miRNAs involved in FMF and their possible mechanisms of action in immunity.

**Table 1:** Role of miRNAs in FMF pathogenesis: expression profile and reported mechanisms of action

miRNAs differentially expressed in FMF patients as compared to controls	Mechanism of actions	Reported role in immunity	Validated role in FMF	Validation experimental approach in FMF	References
↑: miR-144-3p, miR-21-5p, miR-4454, and miR-451a ↓: miR-107, let-7d-5p, and miR-148b-3p	Inflammation	Let-7d-5p represses the proliferation of Th1 cells and IFN- γ release [79]	N/A	N/A	Amarilyo et al. (2018) [93]
		miR-107 acts on CDK6. Upon LPS stimulation, miR-107 is downregulated which promotes the expression of CDK6 and adhesion of macrophages [115]	N/A	N/A	
		miR-148b-3p suppresses the production of IL-12, IL-6, and TNF-α [95]	N/A	N/A	
↑: miR-34a-5p, miR-142-3p, miR-216a-5p, miR-340-5p, miR-429, and miR-582-5p ↓: miR-107, miR-569, and miR-1304-5p	Inflammation	miR-34a-5p activates nuclear factor erythroid-2 related factor 2/heme oxygenase-1 (Nrf2/HO-1) pathway and reduces inflammation [116]	N/A	N/A	Kahraman et al. (2021) [97]
↓ miR-204-3p	Inflammation	IGFBP2/AKT/Bcl2 pathway is regulated by miR-204-3p which suppresses the growth of hepatocellular carcinoma tumor endothelial cells [117]	miR-204-3p inhibits the PI3Kγ pathway which usually promotes the release of inflammatory cytokines including IL-12p40 and IL-6 `	-Transfection of macrophages generated from THP-1 cells with pre-miRNA followed by LPS stimulation -Quantification of inflammatory cytokine production using multiplex cytokine assay -Targeted genes were identified by overexpressing the miRNA and conducting a cDNA microarray -miR-204-3p suppression of PI3K-γ was confirmed by luciferase activity assay	Koga et al. (2017) [98]
↑: miR-20a-5p (in homozygous patients) and let-7d-3p and miR-574-3p (in heterozygous patients) ↓: miR-197-3p (in homozygous patients)	Inflammation	The 4 miRNAs, miR-20a-5p, let-7d-3p, miR-574-3p, and miR-197-3p, were shown to be involved in TGF-β, TLR, and NLR signaling pathways, apoptosis, and actin cytoskeleton regulation [91]	miR-197-3p was proved to repress the expression of *IL1-R1* resulting in the decrease of IL-1β expression and production in FMF patients	miR-197-3p anti-inflammatory role was validated by: - pre-miR-197 transfection -Inflammation related functional assays, apoptosis assay, cell migration assay -Confirmation via anti-miR-197 transfection -Targeting genes studied via 3’UTR luciferase activity assay	Akkaya-Ulum et al. (2017,2021) [91, 101]
		In rheumatoid arthritis, miR-20a was reported to have an anti-inflammatory role by regulating the expression of apoptosis signal-regulating kinase (ASK) 1 which is involved in TLR4 pathway particularly upstream of p38 mitogen-activated protein kinase. Additionally, mir-20 a was found to target signal-regulatory protein α (SIRPα) involved in the regulation of leukocyte inflammatory responses [118, 119]			
		In hepatitis B virus infection, miR-197 was shown to promote liver inflammation by acting on IL-18 [120] In hepatocellular carcinoma, miR-107 was shown to target the IL-6/STAT3 inflammatory signaling pathway [121]			
↓: miR-181a and miR-125a	Inflammation	miR-181a is involved in the differentiation and activation of T cells [110]	N/A	N/A	AbdelKawy et al. (2021) [109]
		miR-125a decreases the production of IL-10 receptor α, IL-2 receptor β, and IFN-γ [109]			
↓: miR-125a, miR-132, miR-146a, miR-155, miR-15a, miR-16, miR-181a, miR-21, miR-223, miR-26a, and miR-34a	Inflammation	mir-155 plays an anti-inflammatory role by inhibiting molecules including matrix metalloproteinases, protein phosphatase 2A, TLR ligands, and IL-2 [103]	N/A	N/A	Hortu et al. (2019) [102]
		mir-223, mir-16, and mir-15a are shown to decrease inflammation by inhibiting the NF- κB pathway in a similar way to NF-κB kinase inhibitors [104]			
		miR-132 has an anti-inflammatory role by affecting the TLR4-NFκB-TNF-a/IL-1β signaling pathway [102]			
		Decrease in mir-125a expression in lupus T cells was associated with an increase in the activity of Kruppel-like factor 13 and an overproduction of the inflammatory chemokine RANTES [122]			
↑: miR-4520a	Autophagy	N/A	miR-4520a targets RHEB which activates mTOR pathway	Using *in silico* and bioinformatic analysis, RHEB was found to be the target of miR-4520a	Latsoudis et al. (2017) [100]
↑: miR-15a-5p, miR-29b-3p, miR-181a-5p, miR-181b-5p, miR-181c-5p, miR-214-3p, and miR-365a-3p ↓: let-7a-5p, let-7c, let-7 g-5p, miR-15b-5p, miR-16-5p, miR-17-5p, miR-23a-3p, miR-24-3p, miR-25-3p, miR-26a-5p, miR-26b-5p, miR-27a-3p, miR-29c-3p, miR-30a-5p, miR-30d-5p, miR-30e-5p, miR-106b-5p, miR-146a-5p, and miR-195-5p	Apoptosis	miR-181b-5p suppresses apoptosis by affecting MEK/ERK/p21 pathway [108]	N/A	N/A	Karpuzoglu et al. (2020) [105]
		Downregulation of miR-17-5p was shown to promote apoptosis by increasing the expression of phosphatase and tensin homolog while the decrease in miR-25 expression was shown to induce apoptosis by upregulating high mobility group box 1 [123, 124]			
		Upregulation of miR-16-5p and miR-195-5p were reported to activate caspases 3 and 9 [106, 107]			

↑: Upregulation; ↓: Downregulation; ASK: Apoptosis signal-regulating kinase; CDK6: Cell division protein kinase 6; IFN-γ: Interferon gamma; IGFBP2/AKT/Bcl2: Insulin-like growth factor-binding protein-2/AKT/B-cell lymphoma 2 y; IL: Interleukin; IL1-R1: Interleukin-1-receptor-1; LPS: Lipopolysaccharide; MEK/ERK/p21: Mitogen-activated protein kinase/extracellular signal-regulated kinase/p21; mTOR: Mammalian target of rapamycin; N/A: Not applicable; NF-κB: Nuclear factor kappa-light-chain-enhancer of activated B cells; NLR: Nod-like receptor; Nrf2/HO-1: Nuclear factor erythroid-2 related factor 2 /heme oxygenase-1; PI3K-γ: Phosphoinositide 3-kinase gamma; RANTES: Regulated upon activation, normal T-cell expressed and secreted; RHEB: Ras homolog enriched in the brain; SIRPα: Signal-regulatory protein α; STAT3: Signal transducer and activator of transcription 3; TGF-β: Transforming growth factor beta; Th1: T helper 1; TLR: Toll-like receptor; TNFα: Tumor necrosis factor alpha

## Conclusion

There is a growing body of evidence indicating that FMF is not a simple monogenic disease and that epigenetics play an important role in the manifestation of this disease. Despite being a recent topic that is currently under deep investigation, epigenetic research has revealed the important role of DNA methylation in FMF and the contributions of several miRNAs to various mechanisms involved in the pathophysiology of the disease, mainly inflammation, apoptosis, and autophagy. These findings suggest the use of epigenetic variations, particularly of miRNAs, in FMF patients as biomarkers and therapeutic tools that may open the door to personalized medicine. However, these studies were performed at very early stages and are associated with various limitations despite their added value. First, the role of histone modifications has not yet been studied in FMF; hence, additional research that focuses on the role of this particular epigenetic mechanism in FMF pathogenesis is needed. Second, most of the studies were restricted by small sample sizes in which participants had similar genotypes, were in the quiescent phase, or were receiving colchicine treatment. Indeed, in future research, a larger and more heterogeneous sample, including patients with different genotypes and during crises, is needed to better understand the inflammatory process underlying FMF. Finally, functional studies exploring the effects and mechanisms of action of colchicine on the different epigenetic mechanisms involved in FMF are essential.

## Data Availability

Not applicable.
